# Systemic Embolism and Septic Shock Complicated Left Atrial Myxoma: Case Report

**DOI:** 10.1155/2009/306375

**Published:** 2010-02-24

**Authors:** B. Trimeche, H. Bouraoui, R. Garbaa, A. Mahdhaoui, M. Ben Rhomdane, S. Ernez-Hajri, G. Jeridi

**Affiliations:** Service de Cardiologie, Hôpital Farhat Hached, 4000 Sousse, Tunisia

## Abstract

Myxoma is the most common primary tumor of the heart. The rarity of infected cardiac myxomas leads to numerous diagnostic and therapeutic difficulties. We present a case of infected left atrial myxoma caused by methicillin-sensible *Staphylococcus aureus* in a 48-year-old woman complicated by systemic embolism and septic shock.

## 1. Introduction

Myxomas are the most common primary tumors of the heart in adults, which have an estimated incidence of 0.5 per million population per year [1]. 

The most common clinical presentation is symptoms of mitral valve stenosis or peripheral embolism. The rarity of infected cardiac myxomas leads to numerous diagnostic and therapeutic difficulties. We report a case of infected left atrial myxoma in a 48-year-old woman complicated by systemic embolism and septic shock.

## 2. Clinical Summary

A 46-year-old woman, presented with subacute dyspnea, maintained fevers for 3 weeks of unkoun origin and fatigue. She had diabetes mellitus and no history of recent surgical or dental intervention or drug abuse.

In the physical examination, the patient appeared ill, cachectic, and tachypneic. She presented a blood pressure of 80/50 mmHg, a heart rate of 120 beats/min and a temperature of 38.2°C. She had jugular venous distention with positive hepatojugular reflux and normal heart sounds without murmurs. Examination of the lungs revealed coarse bilateral breath sounds with inspiratory basal crackles. Abdominal examination revealed no organomegaly; extremities had mild bilateral edema. The remaining examination showed no abnormalities.

Electrocardiogram showed sinus tachycardia with an incomplete right bundle-branch block. Chest radiography was normal. Hematologic laboratory values revealed anemia (haemoglobin: 9 g/dL), leukocytosis white blood cell count: 23 000/mm^3^, an erythrocyte sedimentation rate (ESR) of 100 mm/h, CRP was 64 mg/L, and urine analysis showed no abnormalities.

**Figure 1:**
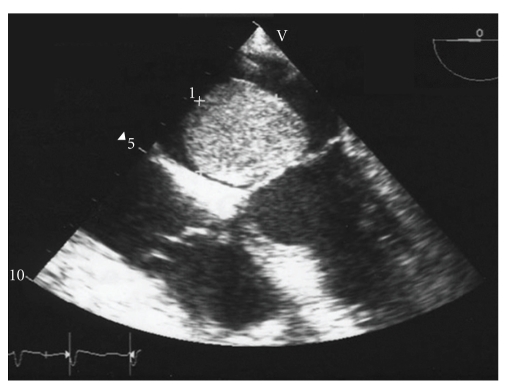
Transesophageal echocardiography revealing large heterogeneous density tumor in the left atrium.

Transthoracic echocardiography and transesophageal echocardiography (Figure 1) revealed a large heterogeneous density tumor in the left atrium, measured about 6 ∗ 5 cm in length adhering to the interatrial septum, with prolapse in the left ventricle. The estimated pulmonary artery pressures were elevated: 82 mmHg. The aortic valve was normal and the mitral valve showed a trace of regurgitation and the left ventricle was hyperkinetic with an estimated ejection fraction of 55%.

Empirical antibiotic therapy with vancomycine and gentamicin was started, surgical treatment was postponed, and blood was drawn for cultures, which were positive for methicillin-sensible *Staphylococcus aureus*. There is no other source of infection; therefore, the atrial mass was suspected to be the cause of sepsis. The patient's hospital course was complicated on day 3 by thrombo embolism cerebral attacks, and then urgent surgical removal was scheduled on the seventh day of hospitalization.

The patient underwent surgical excision of the mass, which was found to be a myxoma infiltrated with abundant inflammatory cells, the cultures of the myxoma which were positive for methicillin-sensible *Staphylococcus aureus*. She died 10 days after surgical intervention by disseminated intravascular coagulation.

## 3. Discussion

Primary cardiac tumors represent less than 0.2% of all neoplasms, three quarters of the tumors are benign, and half of these are myxomas [2]. Myxomas are more common among women and can affect both atria, the ventricles, or the mitral valve; the left atrium is most commonly involved [3, 4].

The most common manifestations are dyspnea and central nervous system embolization; whereas Dias et al. [5] reported peripheral embolism to be dominant. An increased incidence of distal embolization, fever, cachexia, Raynaud phenomenon, or signs of mitral stenosis was noticed by Pinede et al. [6].

 Although there have been several reports of infected myxomas in the recent years, they remain a rare entity [7–10]; a recent literature review, revealed 40 definitive cases of infected myxomas [11].

They can be challenging to diagnose because of their rare occurrence and varied clinical presentation in fact infected and uninfected myxomas; endocardiac thrombus and endocarditis may exhibit the same symptoms (fever, weight loss, fatigue, and malaise) making the correct diagnosis difficult. These systemic symptoms in myxoma, anemia, and raised ESR could be due to the systemic effects of interleukin 6, the cytokine implicated in generating a generalized inflammatory response in patients with myxomas, with levels decreasing after tumor excision [1, 12, 13], then criteria have been proposed by Revankar and Clark [14] to aid in the diagnosis of infected myxoma and they stated that the diagnosis is certain in the presence of myxoma documented by histology, and microorganisms observed in the sample, or a positive blood cultures and evidence of inflammation in the sample. 

In our case, the documented myxoma as evidenced by clinical examination, pathology, and positive blood cultures qualifies this case as a “definite” infected cardiac myxoma. The microorganisms involved were *Streptococcus viridians* (44%) and *Staphylococcus aureus* (15%), a microbiological spectrum similar to that of native valve endocarditis [15].

Systemic embolization occurs in 30% to 40% of patients with myxomas; infected cardiac myxomas are more dangerous than noninfected myxomas and their incidence of embolization is increased two- to threefold [14, 16]. 

Little is known of the correct strategy for treatment of an infected myxoma; surgical excision of cardiac myxoma carries a low operative risk and gives excellent short-term and long-term results. Surgical excision of the tumor appears to be curative, with few recurrences at long-term followup. After diagnosis, surgery should be performed urgently, in order to prevent complications such as embolic events or obstruction of the mitral orifice. 

But there is a dilemma between the urgency to prevent the embolic complications and the need for a surgery on sterile tumor and it seems advisable to recommend surgery despite active infection in an attempt to prevent a catastrophic embolization [8, 9, 17]. 

We conclude that infective left atrial mxoma is an extremely rare condition. The diagnosis is difficult, and because it has such a high incidence of embolization, emergency surgery should be done to remove this type of myxoma once it is diagnosed.
